# A rare tumour of pancreas—Case report

**DOI:** 10.1016/j.ijscr.2020.04.047

**Published:** 2020-05-29

**Authors:** P. Sai Krishna, A. Sivasankar

**Affiliations:** Department of Surgical Gastroenterology, GMKMCH, Salem, India

**Keywords:** Pancreatic schwannoma, Case report, Imaging, Ultrasonography

## Abstract

**Introduction:**

Pancreatic schwannomas are very rare neoplasm arising from Schwann cells that present in the nerve sheath. Schwannoma usually occur in the extremities but can also be found in the trunk, head and neck, retroperitoneum, mediastinum, pelvis and rectum.

**Presentation of case:**

We briefly describe a 30-year-old female patient with upper abdominal pain and on examination by ultrasound and Computed tomography of abdomen revealed a solid tumor arising from body of the pancreas. A laparoscopic splenic vessel preserving distal pancreatectomy (kimura) was performed.

**Discussion:**

Approximately only 50 cases reported in the literature and none of these cases were diagnosed as schwannoma preoperatively. CT scan is often useful to establish pancreatic lesion which usually demonstrate hypodense lesion with encapsulation

**Conclusion:**

Although the incidence of pancreatic schwannoma is low, it must be considered as one possibility in the differential diagnosis of mass lesions in the pancreas and whenever possible especially in large tumors surgical resections should be considered due to increased chance of transformation to malignancy.

## Highlights

• Pancreatic schwannomas are very rare neoplasm arising from Schwann cells that present in the nerve sheath.
• Approximately only 50 cases reported in the literature.
• Although the incidence of pancreatic schwannoma is low, it must be considered as one possibility in the differential diagnosis of mass lesions in the pancreas.
• Whenever possible especially in large tumors surgical resections should be considered as an option due to increased chance of transformation to malignancy.

## 1 Introduction

Pancreatic schwannomas are rare neoplasms that originate from Schwann cells.

Schwannoma is often detected in the head and neck, extremities, mediastinum, and retroperitoneum; however, it is very rare in the pancreas [1].

These tumors vary considerably in size and approximately two-thirds are reported to undergo degenerative changes including cyst formation, calcification, hemorrhage, hyalinization and xanthomatous infiltration [2].

As a result, they may radiographically mimic cystic pancreatic lesions (e.g., mucinous cystic neoplasms, solid and pseudopapillary neoplasms, serous cystic neoplasms, and pseudocysts).

Less than 50 cases of pancreatic schwannoma have been described in the English literature over the past thirty years.

The behavior of this tumor is not clear which can show sometimes malignant degeneration.

Immunohistochemically schwannomas are strongly positive for S-100.

In this report, we present a case of pancreatic schwannoma and provide a pertinent review of literature with emphasis on clinical presentation, diagnosis and treatment done for this case. Work has been reported in line with the SCARE criteria [3].

## 2 Case report

**Fig. 1:**
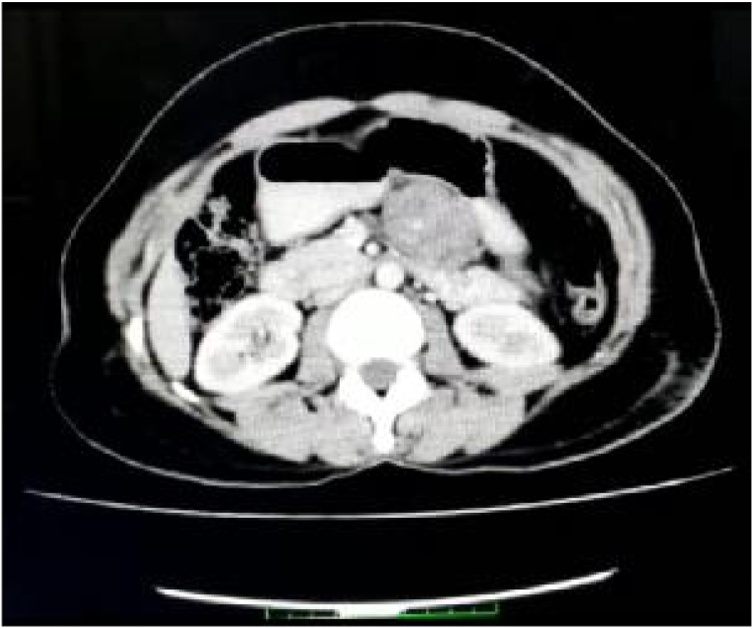
Contrast enhanced CT abdomen showing mass arising from body of pancreas with heteregenous enhancement and central specks of calcification.

**Fig. 2:**
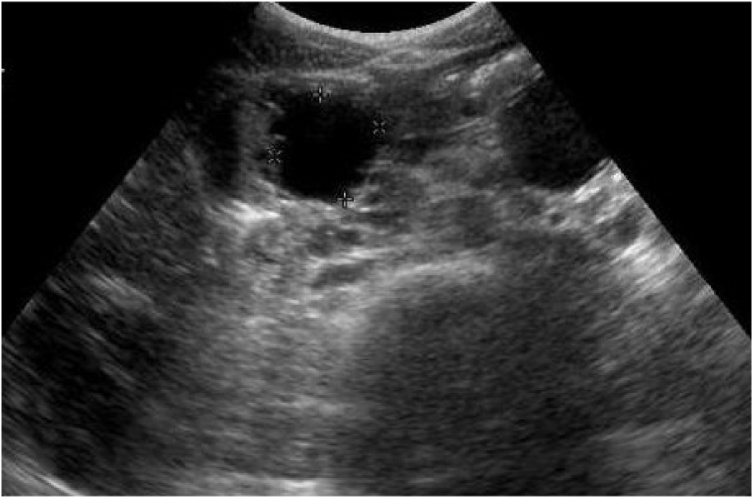
Ultrasonogram of abdomen showing well defined hypoechoic mass arising from body of pancreas.

A 30 year old female came with epigastric pain radiating to upper back Laboratory values including amylase and tumour markers were in normal range. On investigating her a 6*3.5 cm solid well defined oval shaped mass with central specks of calcification arising from the body of the pancreas is identified (Fig. 1, Fig. 2).

**Fig. 3:**
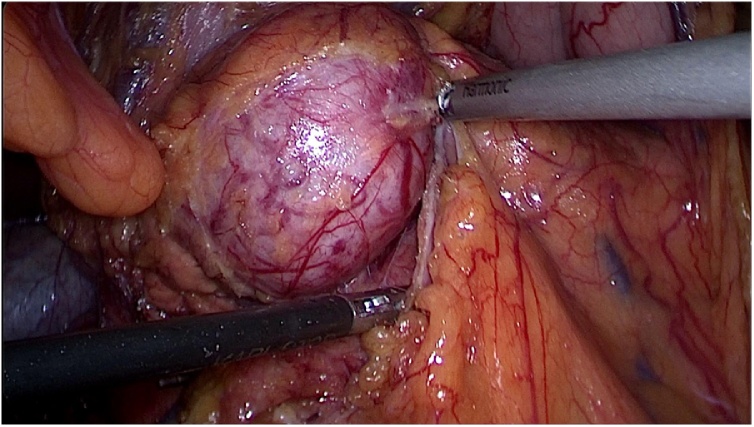
Intra operative – well defined solid mass arising from the anterior surface and inferior border of body portion of pancreas.

**Fig. 4:**
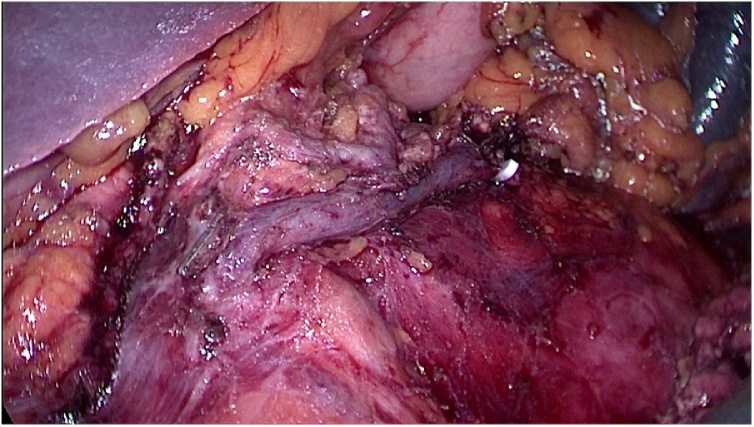
Laparoscopic splenic vessel preserving distal pancreatectomy was performed.

Intra-operatively a well defined solid mass arising from the anterior surface and inferior border of body portion of pancreas was noted (Fig. 3). Examination of other organs and lymph nodes was normal. Laparoscopic splenic vessel preserving distal pancreatectomy (Kimura procedure) was done (Fig. 4).

## 3 Discussion

Pancreatic schwannoma arise from either autonomic sympathetic or para sympathetic fibres, both of which course through pancreas via vagus [4].

In 1910, Verocay reported a schwannoma as a true neoplasm which originated from Schwann cells, and which did not contain neuroganglion cells [5].

**Fig. 5:**
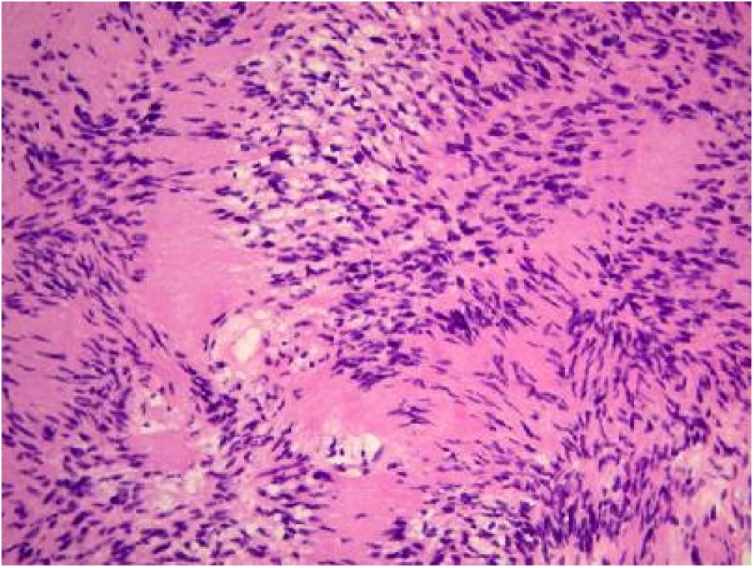
HPE- Unencapsulated neoplasm composed of hypercellular areas containing fascicles of round to oval cells and spindle cells with indistinct cytoplasm and hypocellular areas hyalinised and myxoid stroma.

**Fig. 6:**
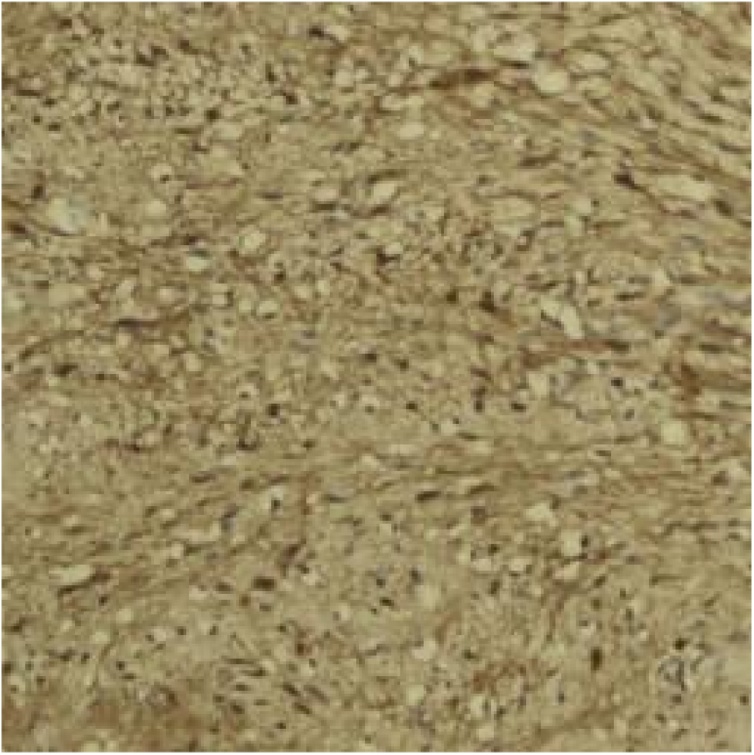
IHC- S100 is positive.

Commonly seen in adults with almost equal male to female ratio. Non-specific abdominal pain is the most commonly reported symptom (Fig. 5, Fig. 6).

Majority of these tumors located in the head (38 %) and body (25 %) of the pancreas [6].

Reported cases of intrapancreatic schwannoma have recently increased in the literature. However, none of these cases were diagnosed as schwannoma preoperatively [7].

CT scan is often useful to establish pancreatic lesion which usually demonstrate hypodense lesion with encapsulation [4,6].

Microscopically, a typical schwannoma is composed of 2 areas, namely Antoni A and Antoni B areas.

In most of the cases, the histological sections of the tumors show the lesion well encapsulated and associated with Antoni A or B patterns, present in a variable proportion [8].

The Antony A pattern has a spindle-long cell which shows a tight fence and higher cells with nuclear double-sequence field (palisading). The Antony B pattern has a loose distribution of the cells with varying degrees of lipoidization [9].

Tumors with a preponderance of Antoni A cells may not appear to be homogeneous due to their high lipid content. Their vascular component gives rise to contrast enhancement.

In tumors with a high level of Antoni B components, poor cellularity and loose stroma may cause a cystic or multiseptated appearance on CT scans [4]. Degenerative or cystic changes such as calcification or hemorrhage are often recognized in the Antoni B area.

These changes result from vascular thrombosis and subsequent necrosis.The MRI findings usually showed hypointensity on T1-weighted images and hyperintensity on T2-weighted images [8].

Immunohistochemically, pancreatic schwannomas are positive for S 100, Vimentin and CD 56. Conversely, spindle cells in pancreatic schwannomas stain negative for cytokeratin, CD117, desmin, CD34, AE1/AE3, alpha smooth muscle actin, and smooth muscle myosin [10].

A review of the treatment in 37 cases revealed that the most commonly performed operation was distal pancreatectomy (10/37) and Whipple procedure (9/37) [2].

Moriya et al., pointed out that malignant formation and cystic formation is strongly associated with increase in tumor diameter [11]. In view of the large tumor size (more than 5 cm) and imaging and intraoperative features of well localised mobile mass arising from body of pancreas with no lymphnode enlargement we choose distal pancreatectomy as a treatment option for this patient. Patient was on regular follow up for the past 3 months and no morbidity noted.

## Declaration of Competing Interest

Authors have no conflict of interest.

## Funding

Nothing to declare.

## Ethical approval

Ethical committee approval given - reference number awaited.

## Consent

Written informed consent was obtained from the patient for publication of this case report and accompanying images. A copy of the written consent is available for review by the Editor-in-Chief of this journal on request.

## Author contribution

Study concept and design - DR. P sai krisshna.

Data collection - DR. P. sai Krishna.

Editing - DR. A. Sivasankar.

## Registration of research studies

Name of the registry:

Unique identifying number or registration ID:

Hyperlink to your specific registration (must be publicly accessible and will be checked):

## Guarantor

Not applicable.
